# Relative Handgrip Strength is Inversely Associated with Hypertension in Consideration of Visceral Adipose Dysfunction: A Nationwide Cross-Sectional Study in Korea

**DOI:** 10.3389/fphys.2022.930922

**Published:** 2022-07-18

**Authors:** Jae Ho Park, Nam-Kyoo Lim, Hyun-Young Park

**Affiliations:** ^1^ Division of Population Health Research, Department of Precision Medicine, Korea National Institute of Health, Korea Disease Control and Prevention Agency, Cheongju, Korea; ^2^ Department of Precision Medicine, Korea National Institute of Health, Korea Disease Control and Prevention Agency, Cheongju, Korea

**Keywords:** hypertension, handgrip strength, muscular strength, visceral adipose dysfunction, visceral obesity

## Abstract

This study investigated the associations of relative handgrip strength (rHGS) and hypertension. Individual differences in visceral adipose dysfunction (VAD) were evaluated to verify whether rHGS was associated with a reduction in the risk of hypertension, even in individuals with VAD. We included 77,991 participants (50,616 women) from nationwide cohorts in Korea. Participants were categorized into three groups based on sex-specific tertiles of rHGS (Low, Mid, and High). The visceral adiposity index (VAI) was used to evaluate VAD. The multiple logistic regression model was used to assess the risk of hypertension. High rHGS is associated with reduction of hypertension risk in 38 and 26% of men and women, respectively, although rHGS was significantly low in women compared to men. The benefit of rHGS was observed from middle-aged to older participants in both sexes. High rHGS is associated with risk reduction for hypertension in both VAD and non-VAD groups. In the VAD group, compared to Low rHGS, High rHGS was associated with 32 and 22% risk reductions in hypertension in men and women, respectively, and these associations remained significant even when classified according to age, such as in middle-aged and older subgroups. Therefore, the present study suggests that high levels of rHGS are significantly associated with a reduced risk of hypertension even in participants with VAD. Thus, maintaining a higher level of rHGS may be associated with protective benefits against hypertension.

## Introduction

The global burden of hypertension has been increasing steadily because of the high regional prevalence and rapid aging of the population in several countries. The World Health Organization (WHO) reported that the prevalence of hypertension has steadily increased and an estimated 1.4 billion persons worldwide have hypertension (World Health Organization, 2021). Hypertension is the leading preventable risk factor for cardiovascular disease (CVD) and premature mortality (Eslami et al., 2019; Fuchs and Whelton, 2020). Risk factors for hypertension, including insulin resistance, dyslipidemia, and obesity, are associated with vascular damage and endothelial dysfunction that are related to an elevated risk of CVD and all-cause mortality as well as micro- and macrovascular complications (Petrie et al., 2018; Yildiz et al., 2020). Thus, preventing and/or treating hypertension seems to be an essential strategy for preventing the abovementioned life-threatening diseases.

Visceral obesity is closely related to the increased risk of hypertension, diabetes mellitus, atherosclerosis, and CVD through mechanisms that increase proinflammatory activity and adipokine production and worsen insulin sensitivity (Després and Lemieux, 2006; Neeland et al., 2019). In contrast, the association of muscular strength with hypertension remains controversial. Aging is associated with reduced muscular strength and muscle mass across the adult lifespan (Keller and Engelhardt, 2013; Haynes et al., 2020). Muscular fitness, which includes muscular strength and muscle mass, has widely been considered as a key factor for healthy aging. Therefore, many studies have investigated the associations between muscular strength and chronic diseases. Assessment of handgrip strength (HGS) is a simple, quick, reliable, and inexpensive method to evaluate muscular fitness in clinical settings (Bohannon, 2008) and is strongly correlated with whole-body muscular strength (Wind et al., 2010; Vaidya and Nariya, 2021). Recent studies have demonstrated inverse and significant associations between HGS and the risk of hypertension (Chon et al., 2020; Feng et al., 2021). However, no significant correlation between HGS and the risk of hypertension was detected in a recent meta-analysis (Bai et al., 2020). Visceral adipose dysfunction (VAD) and visceral obesity increase with aging (Mancuso and Bouchard, 2019; Reyes-Farias et al., 2021) and are positively correlated with hypertension (Ding et al., 2015; Yang et al., 2020). In view of the abovementioned relationship between aging-related processes and VAD or muscular strength, it is important to investigate whether a high HGS level confers protective effects against hypertension prevalence even in participants with VAD. However, to the best of our knowledge, few studies of the correlation between HGS levels and risk of hypertension have been undertaken specifically in participants with VAD. Moreover, investigations of the risk of hypertension by simultaneously considering both muscular strength levels and visceral obesity are lacking.

Therefore, the aim of the present study was to investigate the associations of HGS levels and hypertension in large nationwide cohorts. Moreover, individual differences in VAD were considered to verify whether HGS was associated with risk reduction in hypertension even in individuals with VAD.

## Materials and Methods

### Study Design, Participants, and Measurement

This cross-sectional study used data from the Korean Genome and Epidemiology Study (KoGES), which was conducted by the Korea National Institute of Health. The KoGES is a large consortium project that consists of six prospective cohort studies and aims to establish comprehensive healthcare guidelines for common complex diseases such as hypertension, diabetes mellitus, metabolic syndrome, obesity, CVD, and cancer (Kim and Han, 2017). In this study, we used data collected for 2003–2013 from the KoGES_Health Examinee (HEXA) study, which included 173,202 urban residents (age 40–79 years), as well as data from the seventh wave of the KoGES_Ansan and Ansung study (2013–2014), which included 5,906 participants (age 51–82 years) living in these areas. Participants in each study underwent face-to-face surveys and physical examinations by trained medical staff. A detailed description of these cohort studies is provided in a previous study (Kim and Han, 2017).

Among the 179,108 participants from the two cohorts, the following participants were excluded from the present study: those with a clinical history of CVD and any cancer (n = 11,757); those with incomplete data on parameters necessary for calculating the visceral adiposity index (VAI) such as the waist circumference, body mass index (BMI), triglyceride (TG), and high-density lipoprotein cholesterol (HDL-C) (*n* = 2,955); those without data of HGS and physical activity levels (*n* = 85,812); and those who had no data available for the covariates (*n* = 593). Overall, 77,991 participants (50,616 women) were included in the final analyses (Supplementary Figure S1). This study was approved by the Institutional Review Board Committee of the Korea National Institute of Health, Korea Disease Control and Prevention Agency (Approval No. 2021-04-02-P-A).

Hypertension was defined based on a previous recorded diagnosis of hypertension by a physician, current use of antihypertensive drugs, systolic blood pressure (SBP) ≥140 mmHg, or diastolic blood pressure (DBP) ≥90 mmHg. Blood pressure (BP) was measured by trained healthcare providers using standard methods. SBP and DBP were defined as the average of two readings for the arm with the highest SBP that was obtained after resting for 5 min in the seated position.

Sociodemographic and health-related factors including age, sex, drinking and smoking habits, education level, total time expended for engaging in leisure-time physical activity (PA-time), regularity of resistance training, BMI, waist circumference, diabetes mellitus, and laboratory parameters were included in our analyses. Drinking and smoking habits were classified as “never,” “former,” and “current.” Education level was divided into elementary school graduate or lower, middle or high school graduate, and college graduate or higher. PA-time was defined as the total time (min/week) that was expended for participating regularly in any sports or exercise to the point of sweating. Regularity of resistance training was defined as participation in any resistance training program for more than 1 day per week.

Anthropometric data, including body weight, height, and waist circumference, were measured by trained healthcare providers using standardized protocols. BMI was calculated as body weight (kg) divided by height (m) squared (kg/m^2^). Blood samples were obtained after overnight fasting for at least 8 h. Furthermore, biochemical assays were performed for total cholesterol (T-Chol), HDL-C, TG, fasting blood glucose (FBG), and glycated hemoglobin (HbA1c). Diabetes mellitus was defined based on a previous diagnosis by a physician, current use of antidiabetic medications, including insulin and oral hypoglycemic agents, FBG ≥126 mg/dl, or HbA1c ≥6.5. A detailed description of the biochemical analysis is available elsewhere (Kim and Han, 2017).

### Assessment of Relative HGS

**FIGURE 1:**
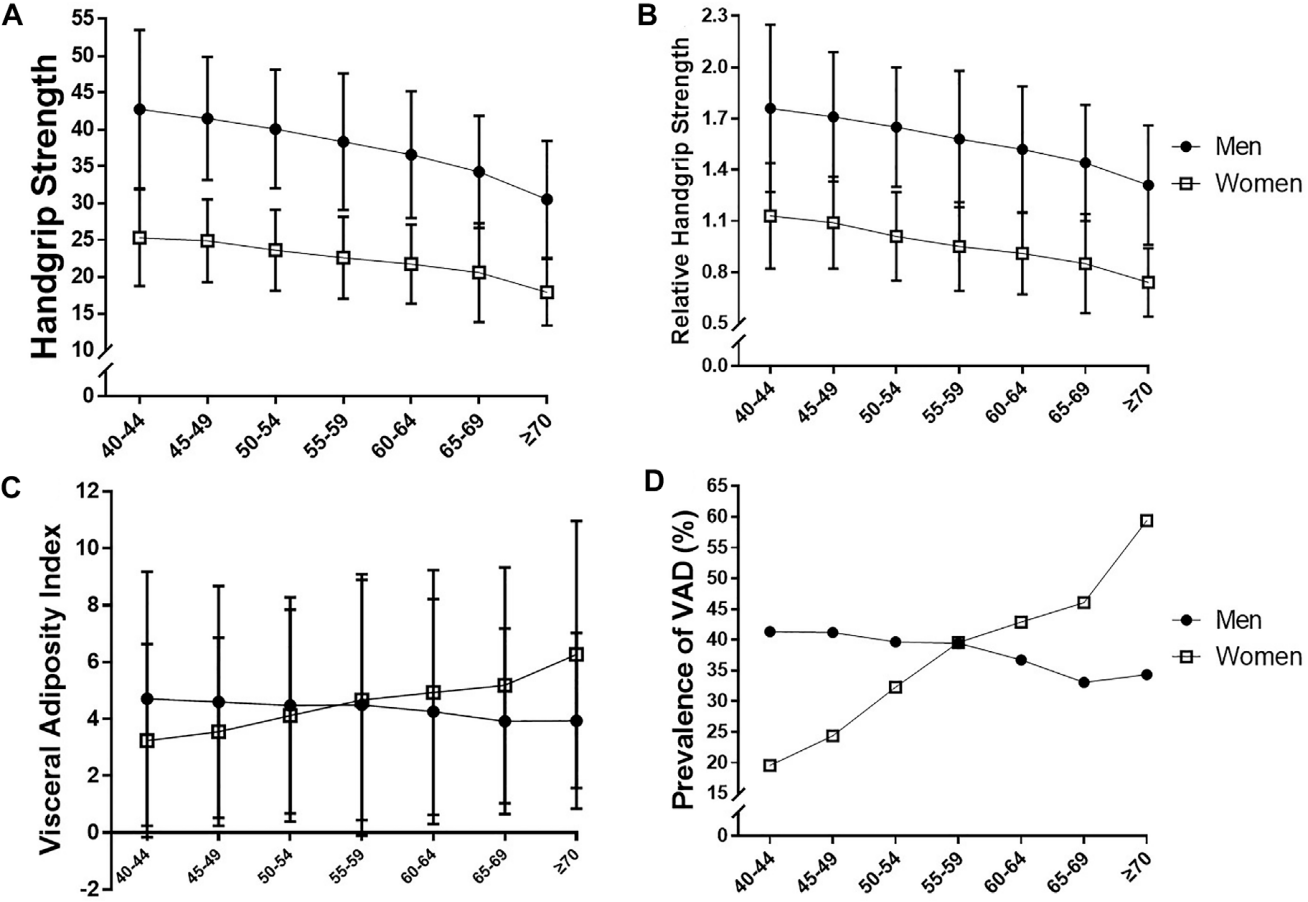
Comparison of **(A)** handgrip strength, **(B)** relative handgrip strength, **(C)** visceral adiposity index, and **(D)** prevalence of visceral adipose dysfunction (VAD) according to age and sex.

The HGS was measured by trained healthcare providers using a digital grip strength dynamometer (T.K.K. 5401; Takei Scientific Instruments Co., Ltd., Tokyo, Japan). During the assessment, participants were instructed to stand upright with their feet apart to hip-width and to look forward, with their elbow fully extended. The dynamometer was held by the testing hand in a comfortable neutral position (not flexed or extended), with 90° of flexion at the index finger. Participants were asked to squeeze the grip continuously, using full force for at least 3 s, and the interval between trials was approximately 60 s. They were encouraged to perform maximally during the tests on a verbal statement: “Squeeze as hard as you can.” HGS was recorded as the mean of two trials of each hand, and the highest reading (in kg) from each hand was used as the absolute HGS. Relative HGS (rHGS) was calculated by dividing the absolute HGS (kg) by BMI (kg/m^2^) as described in previous studies (Yi et al., 2018; Lombardo et al., 2021). Figure 1 presents the age- and sex-stratified levels of HGS and rHGS, and participants were then categorized into their sex-specific tertiles of rHGS (Low, Mid, and High).

### Definition of VAD

VAD was defined based on the VAI which is a sex-specific mathematical index. Waist circumference, BMI, TG, and HDL-C were used to calculate VAI according to the following formula, when TG and HDL-C levels are expressed in millimoles per liter (mmol/L) (Amato et al., 2010): for males, VAI = [waist circumference/39.68 + (1.88 × BMI)] × (TG/1.03) × (1.31/HDL-C); for females, VAI = [waist circumference/36.58 + (1.89 × BMI)] × (TG/0.81) × (1.52/HDL-C). According to the VAI cut-off points for identifying VAD, which were identified as 4.11 in men and 4.28 in women a recent study (Baveicy et al., 2020). Participants were divided into two groups: “Non-VAD” (those without VAD) and “VAD” (those with VAD).

### Statistical Analysis

All statistical analyses were conducted using SAS 9.4 (SAS Institute, Cary, NC, United States). Participant characteristics are presented as descriptive statistics. Continuous variables are presented as means ± standard deviations, whereas categorical variables are expressed as numbers and percentages (%). The chi-square test was used to compare drinking and smoking habits, education levels, and the prevalence of hypertension and diabetes mellitus between groups. Independent t-tests were used to compare age, PA-time, BMI, waist circumference, BP, T-Chol, HDL-C, TG, FBG, and VAI between the groups.

A multiple logistic regression model was used to determine odds ratios (ORs) and 95% confidence intervals (CIs) for the prevalence of hypertension. Models were adjusted for age, sex, drinking, smoking, education level, T-Chol, diabetes mellitus, VAI, and PA-time. All tests were two-tailed, and statistical significance was set at a *p*-value < 0.05.

## Results

Table 1 presents the general characteristics of study participants. The prevalence rates for hypertension in our study population were 35.32 and 25.53% in men and women, respectively. The mean age was slightly higher in men than in women. The prevalence of a high educational level (≥college), current drinking and smoking, regularity of resistance training, VAD, and diabetes mellitus was higher in men than in women. Compared to women, men had significantly higher PA-time, HGS, rHGS, BMI, waist circumference, SBP, DBP, TG, FBG, and VAI, but low T-Chol and HDL-C.

**TABLE 1:** Characteristics of study participants.

Variables	Men (*n* = 27,375)	Women (*n* = 50,616)	*p*-value
Age (years)	53.65 ± 8.88	52.80 ± 8.29	<0.0001
Education level, n (%)	-	-	<0.0001
≤Elementary school	2,444 (8.93)	9,453 (18.68)	-
Middle/high school	14,128 (51.61)	30,306 (59.87)	-
≥College	10,803 (39.46)	10,857 (21.45)	-
Drinking habit, n (%)	-	-	<0.0001
Never drinker	5,118 (18.70)	33,037 (65.27)	-
Ex-drinker	1,733 (6.33)	947 (1.87)	-
Current drinker	20,524 (74.97)	16,632 (32.86)	-
Smoking habit, n (%)	-	-	<0.0001
Never smoker	6,950 (25.39)	48,642 (96.10)	-
Ex-smoker	11,268 (41.16)	678 (1.34)	-
Current smoker	9,157 (33.45)	1,296 (2.56)	-
PA-time (min/week)	186.91 ± 267.78	150.94 ± 223.86	<0.0001
Resistance training, n (%)	3,838 (14.02)	4,112 (8.12)	<0.0001
HGS (kg force)	39.12 ± 9.47	23.37 ± 6.05	<0.0001
rHGS (HGS/BMI)	1.62 ± 0.41	1.01 ± 0.29	<0.0001
BMI (kg/m^2^)	24.41 ± 2.78	23.60 ± 3.01	<0.0001
Waist circumference (cm)	85.57 ± 7.63	78.25 ± 8.41	<0.0001
SBP (mmHg)	125.39 ± 14.21	120.90 ± 15.07	<0.0001
DBP (mmHg)	78.37 ± 9.75	74.41 ± 9.63	<0.0001
T-Chol (mg/dl)	194.06 ± 34.62	199.68 ± 35.60	<0.0001
HDL-C (mg/dl)	49.55 ± 12.01	56.39 ± 13.16	<0.0001
TG (mg/dl)	153.03 ± 109.43	113.90 ± 75.33	<0.0001
FBG (mg/dl)	98.95 ± 24.17	92.90 ± 18.20	<0.0001
VAI	4.43 ± 4.10	4.17 ± 3.90	<0.0001
VAD, n (%)	10,649 (38.90)	16,557 (32.71)	<0.0001
Diabetes mellitus, n (%)	3,678 (13.44)	4,132 (8.16)	<0.0001
Hypertension, n (%)	9,670 (35.32)	12,922 (25.53)	<0.0001

PA-time, total time expended for participating regularly in any sports or exercise to the point of sweating; HGS, handgrip strength; rHGS, relative handgrip strength; BMI, body mass index; SBP, systolic blood pressure; DBP, diastolic blood pressure; T-Chol, total cholesterol; HDL-C, high-density lipoprotein cholesterol; TG, triglycerides; FBG, fasting blood glucose; VAI, visceral adiposity index; VAD, visceral adipose dysfunction.

Figure 1 presents the age- and sex-stratified comparison of HGS, rHGS, VAI, and the prevalence of VAD. There were steady decreases in HGS and rHGS with aging in our study population, although HGS and rHGS were significantly low in women in any age groups compared to men. VAI increased in women with aging, whereas there was no significant change in men. The prevalence of VAD sharply increased in women with aging, and the prevalence of VAD in women exceeded that of men after age 60.

The general characteristics of study participants based on sex-specific rHGS are shown in Supplementary Table S1. In both sexes, compared to the lower rHGS groups, the High rHGS group was younger and significantly related to lower BMI, waist circumference, VAI, SBP, DBP, T-Chol, TG, FBG, and the prevalence of a low educational level (≤elementary school), diabetes mellitus, VAD, and hypertension. However, in both sexes, the High rHGS group markedly related to higher regularity of resistance training, HDL-C, and prevalence of current drinking and smoking, compared to the lower rHGS groups.

**FIGURE 2:**
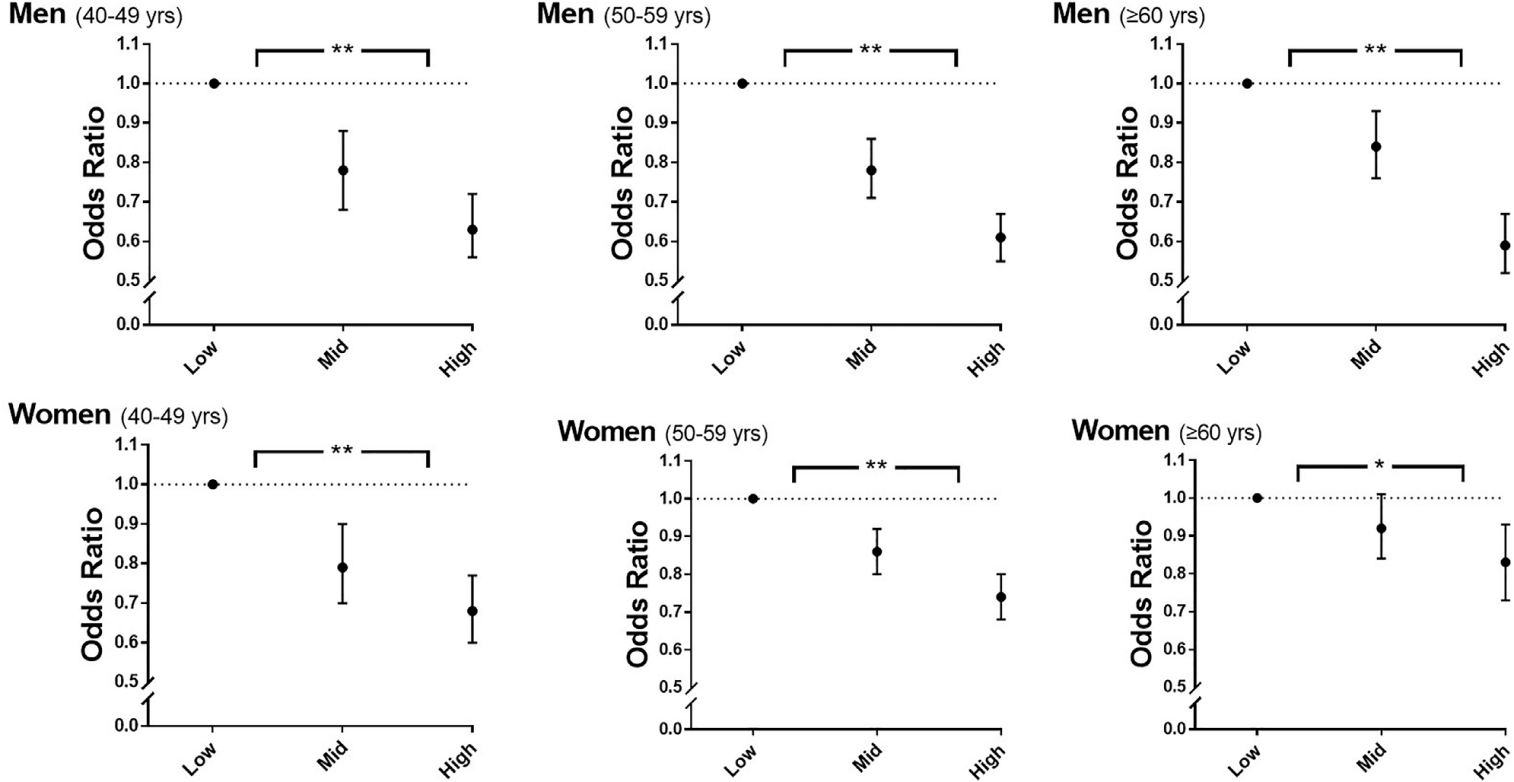
Odds ratios for hypertension according to sex-specific tertiles of rHGS and age. Adjusted for age, drinking, smoking, education level, total cholesterol, diabetes mellitus, visceral adiposity index, and PA-time. rHGS, relative handgrip strength; PA-time,. total time (min/week) expended for participating regularly in any sports or exercise to the point of sweating; yrs, years; *, *p* < 0.001 in the test for trend of ORs; **, *p* < 0.0001 in the test for trend of ORs.

Table 2 shows the inverse relationship between sex-specific tertiles of rHGS and the risk of hypertension after adjustment for covariates (all *p* for trend < 0.0001). In men, compared to participants in Low rHGS, those in Mid and High rHGS were associated with 20 and 38% risk reduction, respectively, for hypertension (all *p* < 0.0001). In women, compared to those in Low rHGS, participants in Mid and High rHGS were associated with 13 and 26% risk reduction of hypertension, respectively (all *p* < 0.0001). These associations remained significant even when classified by age groups as follows: 40–49, 50–59, and ≥60 years (Figure 2). VAD significantly increased the risk of hypertension in both sexes after adjustment for covariates (all *p* < 0.0001; see Supplementary Table S2).

**TABLE 2:** Odds ratios for hypertension according to sex-specific tertiles of rHGS.

Group (rHGS range)	N	Hypertension (%)	rHGS (HGS/BMI)	OR (95% CI)
Men				
Low (≤1.46)	9,125	44.64	1.23 ± 0.21	1 (References)^a^
Mid (1.46–1.75)	9,125	35.46	1.61 ± 0.08	0.80 (0.75–0.85)^*^
High (≥1.75)	9,125	25.87	2.02 ± 0.39	0.62 (0.58–0.66)^*^
Women				
Low (≤0.89)	16,872	35.26	0.73 ± 0.13	1 (References)^a^
Mid (0.89–1.10)	16,872	24.89	1.00 ± 0.06	0.87 (0.83–0.92)^*^
High (≥1.10)	16,872	16.44	1.29 ± 0.26	0.74 (0.70–0.79)^*^

rHGS, relative handgrip strength; HGS, handgrip strength; BMI, body mass index; OR, odds ratio; CI, confidence interval; T-Chol, total cholesterol; PA-time, total time (min/week) expended for participating regularly in any sports or exercise to the point of sweating; ^a^, *p* < 0.0001 in the test for trend of ORs; ^*^, *p* < 0.0001. Adjusted for age, drinking, smoking, education level, T-Chol, diabetes mellitus, visceral adiposity index, and PA-time.

**FIGURE 3:**
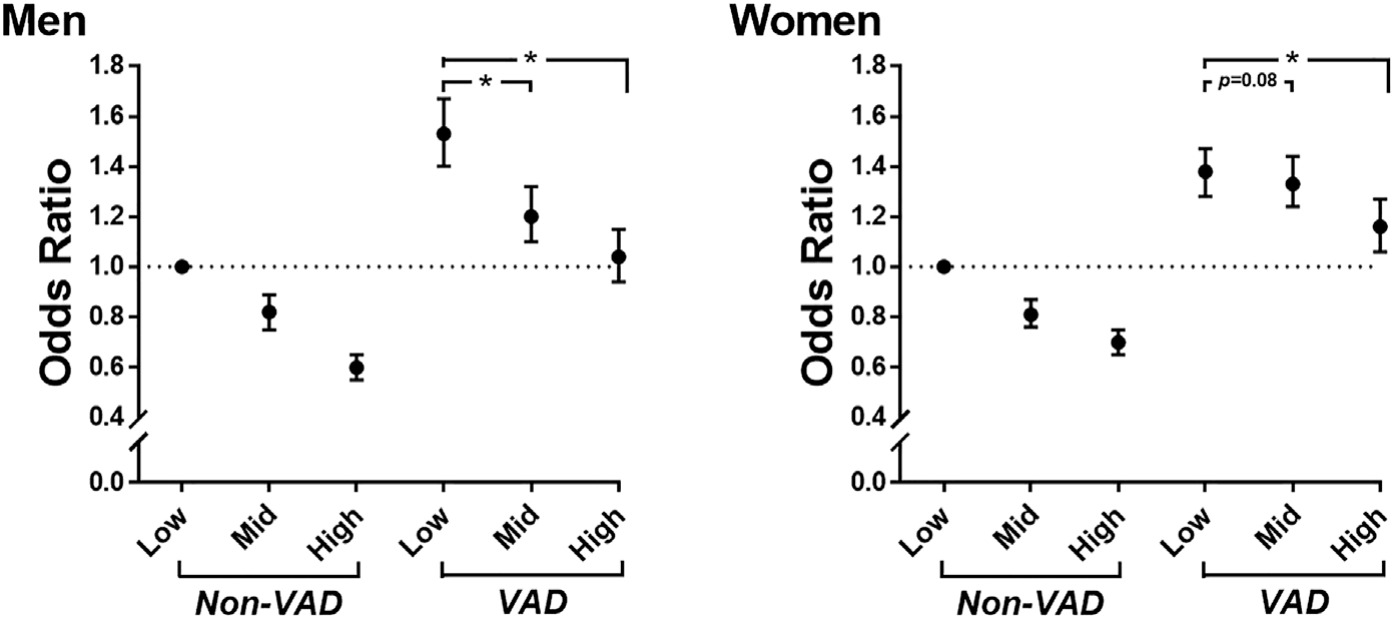
Odds ratios for hypertension according to sex-specific tertiles of rHGS and VAD. Adjusted for age, drinking, smoking, education level, total cholesterol, diabetes mellitus, and PA-time. rHGS, relative handgrip strength; VAD, visceral adipose dysfunction; PA-time, total time (min/week) expended for participating regularly in any sports or exercise to the point of sweating; *, *p < 0.0001.*

We further investigated whether rHGS was associated with risk reduction of hypertension after adjustment with covariates even in participants with VAD. The participants were divided into six subgroups based on their sex-specific tertiles of rHGS and VAD. As shown in Figure 3 and Supplementary Table S3, in men, compared to those in the Low rHGS, male participants in the Mid and High rHGS groups were associated with 18 and 40% risk reduction of hypertension, respectively, in those without VAD (all *p* < 0.0001), whereas those in the Mid and High rHGS groups were associated with 21 and 32% risk reduction of hypertension, respectively, even in those with VAD (all *p* < 0.0001). In women, compared to the Low rHGS group, female participants in the Mid and High rHGS groups were associated with a 19 and 30% risk reduction of hypertension, respectively, in those without VAD (all *p* < 0.0001), whereas only those in the High rHGS were associated with a 22% risk reduction of hypertension in those with VAD (*p* < 0.0001). These associations remained significant even when classified according to the following age groups: 40–49, 50–59, and ≥60 years (Supplementary Figure S2; Supplementary Table S4).

## Discussion

The present study indicated that high levels of rHGS may be associated with protective benefits against hypertension, even in participants with VAD. The abovementioned association remained significant even when classified according to age, such as in the middle-aged and older participant groups. Moreover, in both sexes, simultaneously having a higher level of rHGS and preventing VAD seems to synergistically decrease the risk of hypertension.

Unlike in aerobic exercise training, which plays a beneficial role on vascular health including BP (Fagard, 2006; Pagonas et al., 2017), the associations between resistance training or muscular strength and the factors related to hypertension are controversial. In several studies, higher muscular strength was associated with a lower risk of hypertension (Maslow et al., 2010; Feng et al., 2021). However, this association between muscular strength and risk of hypertension was no longer significant after adjusting for cardiorespiratory fitness (Maslow et al., 2010), and no significant correlation was found between muscular strength and risk of hypertension in a recent meta-analysis (Bai et al., 2020). Moreover, an epidemiological study showed that increased muscular strength was related to higher risk of hypertension as well as DBP in overweight and obese men (Ji et al., 2018). In our study, there was an inverse association between the sex-specific tertiles of rHGS and risk of hypertension even after adjusting for VAI and PA-time, as shown in Table 2. This result supports previous findings of an inverse association between muscular strength and risk of hypertension (Feng et al., 2021). However, considering these contradictory results, it can be inferred that the associations between muscular strength and risk of hypertension have not been fully investigated. Furthermore, the effects of resistance training on the factors related to hypertension are less clearly elucidated and remain controversial. Based on several meta-analyses, resistance training significantly lowers both SBP and DBP regardless of the duration and training intensity (Cornelissen and Smart, 2013), and progressive resistance training (from low- or middle-intensity to high-intensity) markedly lowered BP even in prehypertensive and hypertensive patients (de Sousa et al., 2017). However, several randomized controlled trials (RCTs) revealed no changes in both SBP and DBP following high-intensity resistance training (Cortez-Cooper et al., 2008; Rossow et al., 2014) and low-intensity isometric handgrip training (Pagonas et al., 2017). Thus, the associations between muscular strength or resistance training and BP or hypertension prevalence have remained unclear in humans. Meanwhile, a potential mechanism for the reduction in BP following resistance training, particularly low-intensity resistance training, has been reported to potentially reduce BP by increasing endothelial nitric oxide-mediated vasodilatory function without changing plasma norepinephrine levels (Figueroa et al., 2019). Taken together, considering the findings of previous studies and our results, improving muscular strength via regular resistance training seems necessary to improve vascular health and prevent hypertension, but there was a steady decrease in the regularity of resistance training with aging in our study population, especially in women, as shown in Supplementary Figure S3. Further studies are necessary to verify the protective role of regular resistance training in hypertension and its potential mechanism of action.

Visceral obesity is a well-known modifiable risk factor for hypertension. Waist circumference has been used for the indirect assessment of visceral obesity, but does not help distinguish between visceral and subcutaneous fat mass. Thus, Amato et al. (2010) developed the VAI, which is based on the waist circumference, BMI, TG, and HDL-C (Amato et al., 2010), that has been considered a surrogate marker of both fat distribution and VAD and has shown positive and significant associations with hypertension, cardiometabolic risk, diabetes mellitus, and CVD events (Amato et al., 2011; Ding et al., 2015; Alkhalaqi et al., 2020; Yang et al., 2020). In our study, compared with the Non-VAD group, participants in the VAD group had a significantly higher risk of hypertension in both sexes after adjustment of covariates. This is consistent with the findings of previous studies which reported positive and significant associations between VAD and the risk of hypertension (Ding et al., 2015; Yang et al., 2020). Excessive visceral fat distribution plays a crucial role in the development of hypertension as well as diabetes mellitus by increasing circulating levels of proinflammatory cytokines such as tumor necrosis factor alpha (TNF-α) and interleukin 6 (IL-6), and by worsening insulin sensitivity along with abnormal glucose regulation (Després and Lemieux, 2006; Lopes et al., 2016; Neeland et al., 2019). Taken together, it seems necessary to proactively establish strategies to prevent visceral obesity or VAD, because VAD is profoundly linked to chronic diseases, such as hypertension and diabetes mellitus, which synergistically increase the risk of life-threatening diseases, including CVD and stroke.

Although a previous study found a positive relationship between HGS levels and hypertension prevalence in overweight and obese men in BMI-stratified analysis (Ji et al., 2018), this parameter does not help to distinguish among skeletal muscle, subcutaneous fat, and visceral fat. Therefore, we additionally considered VAD to investigate the association of hypertension prevalence with muscular strength and visceral obesity. In our study, high levels of rHGS significantly lowered the risk of hypertension even in participants with VAD. For women especially, there were steady increases in the prevalence of VAD with aging, and the prevalence in women exceeds men after age 60. Although rHGS was significantly low in women compared to men in any age group, the inverse associations between rHGS and risk of hypertension in those with VAD remained significant regardless of age or sex. Taken together, maintaining a higher level of muscular strength may confer protective benefits against hypertension even in participants with VAD, and this inference is consistent with the findings of several previous RCTs. Resistance training markedly decreased central SBP and DBP as well as brachial SBP and DBP, independently of weight loss and changes in waist circumference and BMI in obese adults (Croymans et al., 2014). In other RCTs, regular resistance training significantly decreased visceral adipose tissue, independently of changes in body mass and BMI in older adults (Ibañez et al., 2005; Yan et al., 2019). More importantly, resistance training can result in significant reductions in the levels of proinflammatory cytokines, such as TNF-α and IL-6, that are produced in adipose tissue independent of weight loss in obese adults and even in patients with diabetes mellitus (Strasser et al., 2012; Miller et al., 2017). Taken together, resistance training may have beneficial effects in the prevention and/or treatment of hypertension by improving visceral obesity which is a causative factor in hypertension or by improving BP even without improvement in obesity. However, for women especially, there were rapid increases in the prevalence of VAD, whereas the regularity of resistance training decreased, with aging in our study population; therefore, it may be necessary to encourage the population to participate in regular resistance training programs for improving muscular strength and for preventing the risk of VAD and hypertension. Considering the cross-sectional nature of our study, further studies are needed to determine the causal associations between resistance training or high levels of muscular strength and the risk of hypertension in participants with visceral obesity or VAD.

The major strength of the present study was the use of large nationwide cohorts that are representative of the Korean general population aged 40–82 years; this makes our results generalizable to the population in this age group. Furthermore, to our knowledge, this is the first study to investigate the risk of hypertension while considering the combined effects of rHGS and VAD. Thus, our results provide further evidence for preventing the risk of hypertension, even in participants with VAD. However, there were several limitations of this study. First, we were unable to deduce causal relationships because of the cross-sectional nature of the present study. Further longitudinal studies are required to unravel the associations that we observed. Second, we used the rHGS as an indicator for muscular strength due to its simplicity and reliability of measurement; however, the rHGS might not represent the whole-body muscular strength or physical function. Moreover, although we investigated the associations between rHGS and hypertension prevalence, we could not calculate a clear cut-off point of rHGS for the prevention of hypertension. Therefore, further studies are needed to clearly verify whether having a higher level of rHGS decreases the risk of hypertension as well as to select an optimal cut-off point of rHGS for mitigating the risk of hypertension in prospective studies. Third, although VAI has been validated by visceral adipose tissue measured by magnetic resonance imaging (Amato et al., 2010; Amato et al., 2011), there exists a likelihood of under- or overestimation of the actual prevalence of VAD. Nevertheless, compared to traditional obesity indicators, including BMI and waist circumference, the VAI has widely been used due to the advantage of being able to simultaneously evaluate both fat distribution and function. Last, our participants comprised exclusively Korean adults; therefore, we cannot be certain whether these results are applicable to other populations.

## Conclusion

The present study suggests that high levels of rHGS may be associated with protective benefits against hypertension, even in participants with VAD, although there was a steady decrease in the rHGS levels with aging in both sexes. Moreover, having a higher level of rHGS and preventing VAD at the same time seems to synergistically decrease the risk of hypertension in both sexes. Taken together, it seems necessary to establish strategies to improve muscular strength and visceral obesity for mitigating the risk of hypertension.

## Data Availability

Publicly available datasets were analyzed in this study. This data can be found here: The data in this study were from the Korean Genome and Epidemiology Study (KoGES; 4851-302), Korea National Institute of Health, Korea Disease Control and Prevention Agency, Korea.
