# Supplementary figures and images for: Relative Handgrip Strength is Inversely Associated with Hypertension in Consideration of Visceral Adipose Dysfunction: A Nationwide Cross-Sectional Study in Korea
Source: Front Physiol. 2022 Jul 18;13:930922. doi: 10.3389/fphys.2022.930922 (PMC9344337; doi:10.3389/fphys.2022.930922)

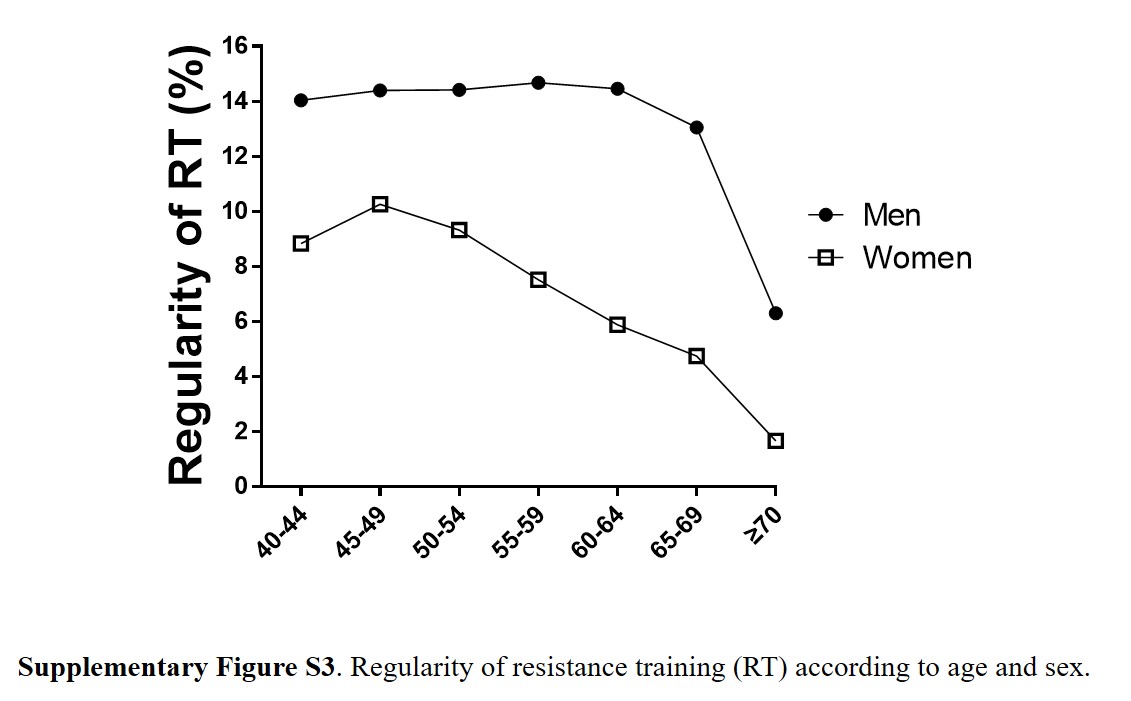

Supplement: Supplementary file 1 [file Image3.jpeg]

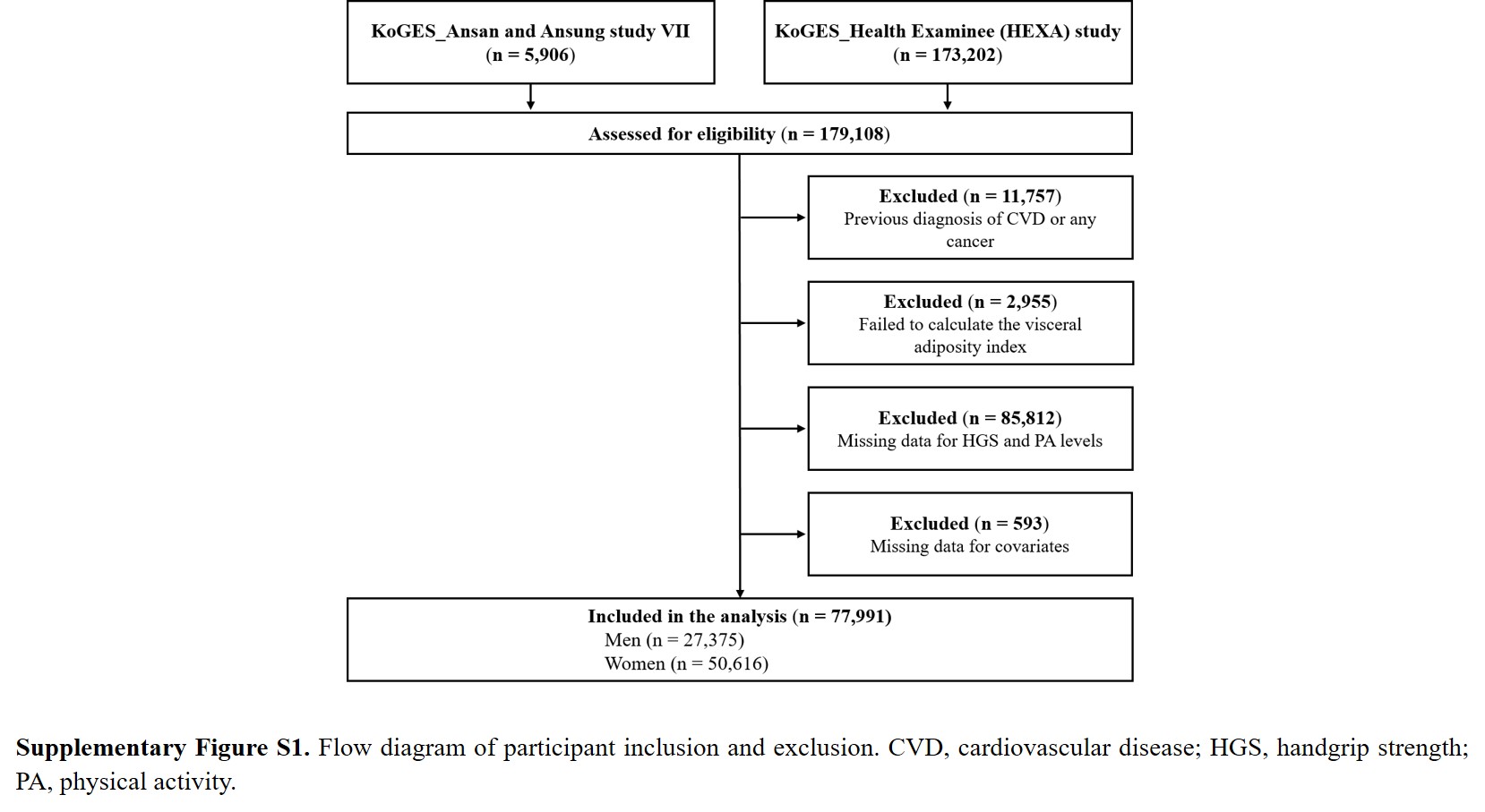

Supplement: Supplementary file 3 [file Image1.jpeg]

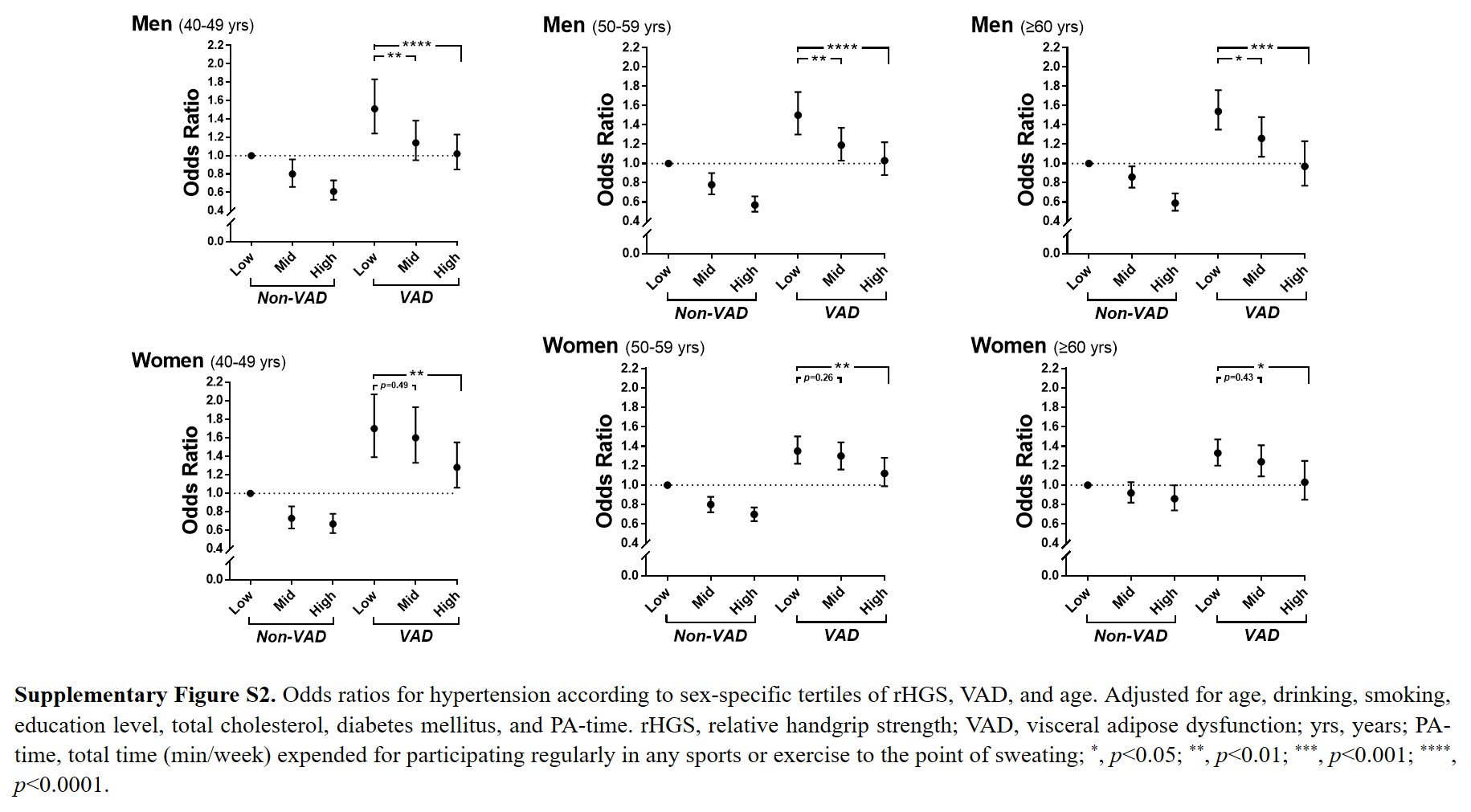

Supplement: Supplementary file 4 [file Image2.jpeg]
