# Supplementary material for: Relative Handgrip Strength is Inversely Associated with Hypertension in Consideration of Visceral Adipose Dysfunction: A Nationwide Cross-Sectional Study in Korea
Source: Front Physiol. 2022 Jul 18;13:930922. doi: 10.3389/fphys.2022.930922 (PMC9344337; doi:10.3389/fphys.2022.930922)
Supplement: Supplementary file 2 [file Table1.docx]

**Supplementary Table S1.** Characteristics of study participants based on sex-specific rHGS

| **Variables** | **Men** (n = 27,375) | | | ***p*-value** | **Women** (n = 50,616) | | | ***p*-value** |
| --- | --- | --- | --- | --- | --- | --- | --- | --- |
|  | **Low**  (n = 9,125) | **Mid**  (n = 9,125) | **High**  (n = 9,125) |  | **Low**  (n = 16,872) | **Mid**  (n = 16,872) | **High**  (n = 16,872) |  |
| **Age** (years) | 56.85 ± 9.10 ^b,c^ | 53.70 ± 8.44 ^a,c^ | 50.41 ± 7.87 ^a,b^ | <0.0001 | 56.46 ± 8.46 ^b,c^ | 52.73 ± 7.70 ^a,c^ | 49.22 ± 7.01 ^a,b^ | <0.0001 |
| **Education level**, n (%) |  |  |  | <0.0001 |  |  |  | <0.0001 |
| ≤Elementary school | 1,205 (13.21) | 772 (8.46) | 467 (5.12) |  | 5,108 (30.27) | 2,857 (16.93) | 1,488 (8.82) |  |
| Middle/high school | 4,391 (48.12) | 4,638 (50.83) | 5,099 (55.88) |  | 9,323 (55.26) | 10,357 (61.39) | 10,626 (62.98) |  |
| ≥College | 3,529 (38.67) | 3,715 (40.71) | 3,559 (39.00) |  | 2,441 (14.47) | 3,658 (21.68) | 4,758 (28.20) |  |
| **Drinking habit**, n (%) |  |  |  | <0.0001 |  |  |  | <0.0001 |
| Never drinker | 1,899 (20.81) | 1,684 (18.45) | 1,535 (16.82) |  | 11,994 (71.09) | 10,955 (64.93) | 10,088 (59.79) |  |
| Ex-drinker | 746 (8.18) | 532 (5.83) | 455 (4.99) |  | 347 (2.06) | 313 (1.86) | 287 (1.70) |  |
| Current drinker | 6,480 (71.01) | 6,909 (75.72) | 7,135 (78.19) |  | 4,531 (26.85) | 5,604 (33.21) | 6,497 (38.51) |  |
| **Smoking habit**, n (%) |  |  |  | <0.0001 |  |  |  | <0.01 |
| Never smoker | 2,502 (27.42) | 2,284 (25.03) | 2,164 (23.71) |  | 16,262 (96.38) | 16,241 (96.26) | 16,139 (95.65) |  |
| Ex-smoker | 3,961 (43.41) | 3,807 (41.72) | 3,500 (38.36) |  | 205 (1.22) | 232 (1.38) | 241 (1.43) |  |
| Current smoker | 2,662 (29.17) | 3,034 (33.25) | 3,461 (37.93) |  | 405 (2.40) | 399 (2.36) | 492 (2.92) |  |
| **PA-time** (min/week) | 183.95 ± 258.45 ^b^ | 193.97 ± 280.37 ^a,c^ | 182.80 ± 263.91 ^b^ | <0.01 | 132.54 ± 205.59 ^b,c^ | 155.55 ± 224.11 ^a,c^ | 164.73 ± 239.39 ^a,b^ | <0.0001 |
| **Resistance training**, n (%) | 1,314 (14.40) ^b^ | 1,186 (13.00) ^a,c^ | 1,338  (14.66) ^b^ | <0.01 | 1,118 (6.63) ^b,c^ | 1,417 (8.40) ^a,c^ | 1,577 (9.35) ^a,b^ | <0.0001 |
| **HGS** (kg force) | 31.32 ± 6.14 ^b,c^ | 39.42 ± 4.29 ^a,c^ | 46.58 ± 9.80 ^a,b^ | <0.0001 | 18.44 ± 3.83 ^b,c^ | 23.51 ± 2.67 ^a,c^ | 28.16 ± 6.38 ^a,b^ | <0.0001 |
| **rHGS** (HGS/BMI) | 1.23 ± 0.21 ^b,c^ | 1.61 ± 0.08 ^a,c^ | 2.02 ± 0.39 ^a,b^ | <0.0001 | 0.73 ± 0.13 ^b,c^ | 1.00 ± 0.06 ^a,c^ | 1.29 ± 0.26 ^a,b^ | <0.0001 |
| **BMI** (kg/m^2^) | 25.53 ± 2.83 ^b,c^ | 24.52 ± 2.50 ^a,c^ | 23.17 ± 2.48 ^a,b^ | <0.0001 | 25.24 ± 3.18 ^b,c^ | 23.59 ± 2.51 ^a,c^ | 21.96 ± 2.30 ^a,b^ | <0.0001 |
| **Waist circumference** (cm) | 88.36 ± 7.57 ^b,c^ | 85.87 ± 6.99 ^a,c^ | 82.50 ± 7.16 ^a,b^ | <0.0001 | 82.39 ± 8.72 ^b,c^ | 78.10 ± 7.41 ^a,c^ | 74.27 ± 6.96 ^a,b^ | <0.0001 |
| **SBP** (mmHg) | 126.57 ± 14.35 ^b,c^ | 125.71 ± 14.21 ^a,c^ | 123.89 ± 13.94 ^a,b^ | <0.0001 | 123.30 ± 15.32 ^b,c^ | 121.05 ± 14.92 ^a,c^ | 118.34 ± 14.54 ^a,b^ | <0.0001 |
| **DBP** (mmHg) | 78.86 ± 9.58 ^c^ | 78.54 ± 9.80 ^c^ | 77.70 ± 9.82 ^a,b^ | <0.0001 | 75.69 ± 9.57 ^b,c^ | 74.56 ± 9.58 ^a,c^ | 72.97 ± 9.55 ^a,b^ | <0.0001 |
| **T-Chol** (mg/dL) | 194.19 ± 35.55 | 195.02 ± 34.51 ^c^ | 192.97 ± 33.73 ^b^ | <0.001 | 202.09 ± 37.14 ^c^ | 201.20 ± 35.49 ^c^ | 195.76 ± 33.77 ^a,b^ | <0.0001 |
| **HDL-C** (mg/dL) | 47.82 ± 11.48 ^b,c^ | 49.28 ± 11.77 ^a,c^ | 51.54 ± 12.48 ^a,b^ | <0.0001 | 53.69 ± 12.50 ^b,c^ | 56.28 ± 12.88 ^a,c^ | 59.18 ± 13.50 ^a,b^ | <0.0001 |
| **TG** (mg/dL) | 161.77 ± 112.39 ^b,c^ | 156.05 ± 109.07 ^a,c^ | 141.28 ± 105.72 ^a,b^ | <0.0001 | 126.87 ± 78.53 ^b,c^ | 115.42 ± 78.90 ^a,c^ | 99.41 ± 65.19 ^a,b^ | <0.0001 |
| **FBG** (mg/dL) | 101.93 ± 27.45 ^b,c^ | 99.03 ± 23.24 ^a,c^ | 95.89 ± 20.98 ^a,b^ | <0.0001 | 95.58 ± 21.43 ^b,c^ | 92.61 ± 17.10 ^a,c^ | 90.50 ± 15.13 ^a,b^ | <0.0001 |
| **VAI** | 4.85 ± 4.22 ^b,c^ | 4.53 ± 4.14 ^a,c^ | 3.91 ± 3.87 ^a,b^ | <0.0001 | 4.90 ± 4.17 ^b,c^ | 4.21 ± 4.06 ^a,c^ | 3.41 ± 3.28 ^a,b^ | <0.0001 |
| **VAD**, n (%) | 4,109 (45.03) ^b,c^ | 3,680 (40.33) ^a,c^ | 2,860 (31.34) ^a,b^ | <0.0001 | 7,219 (42.79) ^b,c^ | 5,570 (33.01) ^a,c^ | 3,768 (22.33) ^a,b^ | <0.0001 |
| **Diabetes mellitus**, n (%) | 1,721 (18.86) ^b,c^ | 1,220 (13.37) ^a,c^ | 737 (8.08) ^a,b^ | <0.0001 | 2,232 (13.23) ^b,c^ | 1,250 (7.41) ^a,c^ | 650 (3.85) ^a,b^ | <0.0001 |
| **Hypertension**, n (%) | 4,073 (44.64) ^b,c^ | 3,236 (35.46) ^a,c^ | 2,361 (25.87) ^a,b^ | <0.0001 | 5,949 (35.26) ^b,c^ | 4,200 (24.89) ^a,c^ | 2,773 (16.44) ^a,b^ | <0.0001 |

rHGS, relative handgrip strength; PA-time, total time expended for participating regularly in any sports or exercise to the point of sweating; HGS, handgrip strength; BMI, body mass index; SBP, systolic blood pressure; DBP, diastolic blood pressure; T-Chol, total cholesterol; HDL-C, high-density lipoprotein cholesterol; TG, triglycerides; FBG, fasting blood glucose; VAI, visceral adiposity index; VAD, visceral adipose dysfunction; ^a^, *p*<0.05 vs. Low-rHGS; ^b^, *p*<0.05 vs. Mid-rHGS; ^c^, *p*<0.05 vs. High-rHGS.
