# Supplementary material for: Relative Handgrip Strength is Inversely Associated with Hypertension in Consideration of Visceral Adipose Dysfunction: A Nationwide Cross-Sectional Study in Korea
Source: Front Physiol. 2022 Jul 18;13:930922. doi: 10.3389/fphys.2022.930922 (PMC9344337; doi:10.3389/fphys.2022.930922)
Supplement: Supplementary file 5 [file Table2.docx]

**Supplementary Table S2.** Odds ratios for hypertension according to VAD and sex

|  | **N** | **Hypertension** (%) | **rHGS** (HGS/BMI) | **OR** (95% CI) |
| --- | --- | --- | --- | --- |
| **Total** |  |  |  |  |
| Non-VAD | 50,785 | 24.20^a^ | 1.24 ± 0.45^a^ | 1 (reference) |
| VAD | 27,206 | 37.86^a^ | 1.18 ± 0.44^a^ | 1.65 (1.60–1.71)^*^ |
| **Men** |  |  |  |  |
| Non-VAD | 16,726 | 31.63^a^ | 1.65 ± 0.42^a^ | 1 (reference) |
| VAD | 10,649 | 41.12^a^ | 1.56 ± 0.40^a^ | 1.63 (1.55–1.72)^*^ |
| **Women** |  |  |  |  |
| Non-VAD | 34,059 | 20.56^a^ | 1.04 ± 0.29^a^ | 1 (reference) |
| VAD | 16,557 | 35.76^a^ | 0.94 ± 0.26^a^ | 1.55 (1.49–1.63)^*^ |
| VAD, visceral adipose dysfunction; rHGS, relative handgrip strength; HGS, handgrip strength; BMI, body mass index; OR, odds ratio; CI, confidence interval; T-Chol, total cholesterol; PA-time, total time (min/week) expended for participating regularly in any sports or exercise to the point of sweating; ^a^, *p*<0.0001 compared Non-VAD with VAD; ^*^, *p*<0.0001. Adjusted for age, sex, drinking, smoking, education level, T-Chol, diabetes mellitus, and PA-time. | | | | |
