# Supplementary material for: Relative Handgrip Strength is Inversely Associated with Hypertension in Consideration of Visceral Adipose Dysfunction: A Nationwide Cross-Sectional Study in Korea
Source: Front Physiol. 2022 Jul 18;13:930922. doi: 10.3389/fphys.2022.930922 (PMC9344337; doi:10.3389/fphys.2022.930922)
Supplement: Supplementary file 6 [file Table3.docx]

**Supplementary Table S3.** Odds ratios for hypertension according to sex-specific tertiles of rHGS and VAD

|  | **N** | **Hypertension** (%) | **rHGS** (HGS/BMI) | **OR** (95% CI) | |
| --- | --- | --- | --- | --- | --- |
|  |  |  |  |  |  |
| **Men** |  |  |  |  |  |
| Low/Non-VAD | 5,016 | 41.19 | 1.23 ± 0.21 | 1 (reference) |  |
| Mid/Non-VAD | 5,445 | 32.78 | 1.61 ± 0.08 | 0.82 (0.75–0.89)^**^ |  |
| High/Non-VAD | 6,265 | 22.98 | 2.03 ± 0.38 | 0.60 (0.55–0.65)^**^ |  |
| Low/VAD | 4,109 | 48.84 | 1.22 ± 0.21 | 1.53 (1.40–1.67)^**^ | 1 (reference) |
| Mid/VAD | 3,680 | 39.43 | 1.61 ± 0.08 | 1.20 (1.10–1.32)^**^ | 0.79 (0.72–0.87)^**^ |
| High/VAD | 2,860 | 32.20 | 2.00 ± 0.41 | 1.04 (0.94–1.15) | 0.68 (0.61–0.76)^**^ |
| **Women** |  |  |  |  |  |
| Low/Non-VAD | 9,653 | 29.84 | 0.74 ± 0.13 | 1 (reference) |  |
| Mid/Non-VAD | 11,302 | 20.45 | 1.00 ± 0.06 | 0.81 (0.76–0.87)^**^ |  |
| High/Non-VAD | 13,104 | 13.81 | 1.29 ± 0.27 | 0.70 (0.65–0.75)^**^ |  |
| Low/VAD | 7,219 | 42.51 | 0.73 ± 0.14 | 1.38 (1.28–1.47)^**^ | 1 (reference) |
| Mid/VAD | 5,570 | 33.91 | 0.99 ± 0.06 | 1.33 (1.24–1.44)^**^ | 0.93 (0.86–1.01) |
| High/VAD | 3,768 | 25.56 | 1.26 ± 0.24 | 1.16 (1.06–1.27)^*^ | 0.78 (0.71–0.85)^**^ |
| rHGS, relative handgrip strength; VAD, visceral adipose dysfunction; HGS, handgrip strength; BMI, body mass index; OR, odds ratio; CI, confidence interval; T-Chol, total cholesterol; PA-time, total time (min/week) expended for participating regularly in any sports or exercise to the point of sweating; ^*^, *p*<0.01; ^**^, *p*<0.0001. Adjusted for age, drinking, smoking, education level, T-Chol, diabetes mellitus, and PA-time. | | | | | |
