# Supplementary material for: Relative Handgrip Strength is Inversely Associated with Hypertension in Consideration of Visceral Adipose Dysfunction: A Nationwide Cross-Sectional Study in Korea
Source: Front Physiol. 2022 Jul 18;13:930922. doi: 10.3389/fphys.2022.930922 (PMC9344337; doi:10.3389/fphys.2022.930922)
Supplement: Supplementary file 7 [file Table4.docx]

**Supplementary Table S4.** Odds ratios for hypertension according to sex-specific tertiles of rHGS, VAD, and age

| **Age** (years) | **N** | **Hypertension** (%) | **rHGS** (HGS/BMI) | **OR** (95% CI) | | | | | |
| --- | --- | --- | --- | --- | --- | --- | --- | --- | --- |
|  |  |  |  | **Non-VAD** | | | **VAD** | | |
|  |  |  |  | Low | Mid | High | Low | Mid | High |
| **Men** |  |  |  |  |  |  |  |  |  |
| 40–49 | 9,553 | 23.39 | 1.74 ± 0.45 | 1 (reference) | 0.80 (0.66–0.96)^*^ | 0.61 (0.52–0.73)^****^ | 1.51 (1.24–1.83)^****^ | 1.14 (0.95–1.38) | 1.02 (0.85–1.23) |
|  |  |  |  | - | - | - | 1 (reference) | 0.75 (0.63–0.89)^**^ | 0.67 (0.56–0.79)^****^ |
| 50–59 | 10,113 | 35.97 | 1.62 ± 0.38 | 1 (reference) | 0.78 (0.68–0.90)^***^ | 0.57 (0.50–0.66)^****^ | 1.50 (1.30–1.74)^****^ | 1.19 (1.03–1.37)^*^ | 1.03 (0.88–1.22) |
|  |  |  |  | - | - | - | 1 (reference) | 0.79 (0.68–0.92)^**^ | 0.70 (0.59–0.83)^****^ |
| ≥60 | 7,709 | 49.27 | 1.47 ± 0.37 | 1 (reference) | 0.86 (0.75–0.97)^*^ | 0.59 (0.51–0.69)^****^ | 1.54 (1.35–1.76)^****^ | 1.26 (1.07–1.48)^**^ | 0.97 (0.77–1.23) |
|  |  |  |  | - | - | - | 1 (reference) | 0.82 (0.69–0.98)^*^ | 0.64 (0.50–0.81)^***^ |
| **Women** |  |  |  |  |  |  |  |  |  |
| 40–49 | 18,383 | 10.52 | 1.11 ± 0.29 | 1 (reference) | 0.73 (0.62–0.86)^***^ | 0.67 (0.57–0.78)^****^ | 1.70 (1.39–2.07)^****^ | 1.60 (1.33–1.93)^****^ | 1.28 (1.06–1.55)^*^ |
|  |  |  |  | - | - | - | 1 (reference) | 0.93 (0.76–1.14) | 0.73 (0.59–0.90)^**^ |
| 50–59 | 20,968 | 27.00 | 0.99 ± 0.26 | 1 (reference) | 0.80 (0.72–0.88)^****^ | 0.70 (0.63–0.77)^****^ | 1.35 (1.22–1.50)^****^ | 1.30 (1.16–1.44)^****^ | 1.12 (0.99–1.28) |
|  |  |  |  | - | - | - | 1 (reference) | 0.94 (0.84–1.05) | 0.80 (0.70–0.92)^**^ |
| ≥60 | 11,265 | 47.29 | 0.87 ± 0.26 | 1 (reference) | 0.92 (0.82–1.03) | 0.86 (0.74–0.999)^*^ | 1.33 (1.20–1.47)^****^ | 1.24 (1.09–1.41)^**^ | 1.03 (0.85–1.25) |
|  |  |  |  | - | - | - | 1 (reference) | 0.95 (0.83–1.08) | 0.79 (0.65–0.96)^*^ |
| rHGS, relative handgrip strength; VAD, visceral adipose dysfunction; HGS, handgrip strength; BMI, body mass index; OR, odds ratio; CI, confidence interval; T-Chol, total cholesterol; PA-time, total time (min/week) expended for participating regularly in any sports or exercise to the point of sweating; ^*^, *p*<0.05; ^**^, *p*<0.01; ^***^, *p*<0.001; ^****^, *p*<0.0001. Adjusted for age, drinking, smoking, education level, T-Chol, diabetes mellitus, and PA-time. | | | | | | | | | |
